# 达雷妥尤单抗治疗异基因造血干细胞移植后复发急性T淋巴细胞白血病1例

**DOI:** 10.3760/cma.j.cn121090-20231130-00285

**Published:** 2024-07

**Authors:** 馨 王, 明明 胡, 金燕 肖, 志洪 林, 婷婷 施, 祥飞 李, 惠英 仇

**Affiliations:** 1 苏州永鼎医院血液内科，苏州 215200 Hygeia Suzhou Yongding Hospital, Suzhou 215200, China; 2 苏州大学附属第一医院血液内科，苏州 215006 Department of Hematology, First Affiliated Hospital of Suzhou University, Suzhou 215006, China

患者，女，32岁，2020年12月因头晕、乏力就诊于当地医院。B超检查示：双侧颈部、腋窝、腹股沟多发淋巴结肿大。血常规：WBC 21.6×10^9^/L，HGB 76 g/L，PLT 107×10^9^/L。骨髓穿刺发现原始幼稚细胞54％，流式细胞术分析示T淋系表达（CD34、CD7、CD13、CD33、TDT、cCD3、CD5、CD99阳性，CD19、cCD79a弱阳性）伴髓系、B淋系表达（CD5 83％，CD99 97％，CD1a 0.3％，cCD79dim 60.3％，MPO 0.3％，cCD3 92.6％，TDT 47.6％），染色体核型为46,XX,6q−,t（10；12）（p15；q23）,12p+[3]/46，XX[4]，多重PCR检查阴性，二代测序检测到NOTCH1突变21.7％、CREBBP突变20.9％、IKZF1突变16.3％、KRAS突变18％、RAD21突变18.4％、SUZ12突变16.6％。诊断为急性T淋巴细胞白血病。患者初始用CIVP（环磷酰胺+伊达比星+长春地辛+地塞米松）方案诱导化疗，获得骨髓完全缓解（CR），微小残留病（MRD）4.4％，染色体核型正常，NOTCH1、CREBBP、IKZF1基因突变均阴性。随后继续予培门冬酶+hyperCVAD（B）方案巩固治疗，骨髓持续CR，MRD<1.5×10^−4^，继续予hyperCVAD（A）方案巩固化疗1次。患者于2021年4月26日接受allo-HSCT（供者胞妹，HLA全相合），预处理方案为改良Bu/Cy方案（司莫斯汀+阿糖胞苷+白消安+环磷酰胺），单个核细胞回输量10.84×10^8^/kg，CD34^+^细胞回输量6.61×10^6^/kg。患者于移植后10 d粒细胞植入，13 d血小板植入。患者移植后未出现明显急/慢移植物抗宿主病（GVHD）。规律复查骨髓均为CR，MRD阴性，细胞短串联重复序列（STR）在98.0％以上。2022年9月21日患者复查骨髓原始细胞占3.5％，MRD 3.8％，STR 93.7％，考虑疾病复发。随后（2022年9月27日）给予供者淋巴细胞输注（DLI）联合干扰素α治疗，治疗后2周骨髓原幼细胞占16.5％，MRD 15.1％，诊断为移植后白血病复发，原幼细胞较前上升，提示DLI疗效不佳。鉴于患者此次复查骨髓提示肿瘤细胞CD38阳性（阳性表达率高达96.3％），于2022年10月予2次达雷妥尤单抗（16 mg/kg，共800 mg）单药治疗（间隔1周），用药过程顺利，未发生不良反应。期间监测患者胆红素指标进行性升高，同时躯干部及四肢出现散在斑丘疹，考虑肝脏、皮肤GVHD，经甲泼尼龙、他克莫司联合芦可替尼抗GVHD后患者皮疹逐步消褪，但胆红素水平仍持续升高，再予巴利昔单抗（共3次）加强抗GVHD、血浆置换（共1次）治疗后胆红素水平明显下降，肝脏GVHD控制。于2022年11月2日复查骨髓形态示原幼细胞占1.0％，MRD<1.0×10^−4^；STR 98.8％，提示本病缓解。1个月后复查患者骨髓细胞形态持续完全缓解，MRD阴性，STR 98.7％，CD38表达3.11％。随访13个月，持续处于CR状态。

